# Dose-Dependent Hepacivirus Infection Reveals Linkage between Infectious Dose and Immune Response

**DOI:** 10.1128/spectrum.01686-22

**Published:** 2022-08-22

**Authors:** André Gömer, Julien Delarocque, Christina Puff, Maximilian K. Nocke, Birthe Reinecke, Wolfgang Baumgärtner, Jessika M. V. Cavalleri, Karsten Feige, Eike Steinmann, Daniel Todt

**Affiliations:** a Department of Molecular and Medical Virology, Ruhr University Bochum, Bochum, Germany; b Institute of Virology, University of Veterinary Medicine Hannover, Hanover, Germany; c Clinic for Horses, University of Veterinary Medicine Hannover, Hanover, Germany; d Department of Pathology, University of Veterinary Medicine Hannover, Hanover, Germany; e Institute of Experimental Virology, TWINCORE Centre for Experimental and Clinical Infection Research, Hanover, Germany; f Clinical Section of Equine Internal Medicine, Department of Companion Animals and Horses, University of Veterinary Medicine Vienna, Vienna, Austria; g European Virus Bioinformatics Center (EVBC), Jena, Germany; Wuhan Institute of Virology

**Keywords:** clearance and persistence, dose-dependent infection, hepacivirus, immune response, minimal infectious dose

## Abstract

More than 70 million people worldwide are still infected with the hepatitis C virus 30 years after its discovery, underscoring the need for a vaccine. To develop an effective prophylactic vaccine, detailed knowledge of the correlates of protection and an immunocompetent surrogate model are needed. In this study, we describe the minimum dose required for robust equine hepacivirus (EqHV) infection in equids and examined how this relates to duration of infection, seroconversion, and transcriptomic responses. To investigate mechanisms of hepaciviral persistence, immune response, and immune-mediated pathology, we inoculated eight EqHV naive horses with doses ranging from 1–2 copies to 1.3 × 10^6^ RNA copies per inoculation. We characterized infection kinetics, pathology, and transcriptomic responses via next generation sequencing. The minimal infectious dose of EqHV in horses was estimated at 13 RNA copies, whereas 6 to 7 copies were insufficient to cause infection. Peak viremia did not correlate with infectious dose, while seroconversion and duration of infection appeared to be affected. Notably, seroconversion was undetectable in the low-dose infections within the surveillance period (40 to 50 days). In addition, transcriptomic analysis revealed a nearly dose-dependent effect, with greater immune activation and inflammatory response observed in high-dose infections than in low-dose infections. Interestingly, inoculation with 6–7 copies of RNA that did not result in productive infection, but was associated with a strong immune response, similar to that observed in the high-dose infections.

**IMPORTANCE** We demonstrate that the EqHV dose of infection plays an important role for inducing immune responses, possibly linked to early clearance in high-dose and prolonged viremia in low-dose infections. In particular, pathways associated with innate and adaptive immune responses, as well as inflammatory responses, were more strongly upregulated in high-dose infections than in lower doses. Hence, inoculation with low doses may enable EqHV to evade strong immune responses in the early phase and therefore promote robust, long-lasting infection.

## INTRODUCTION

Thirty years ago, the hepatitis C virus (HCV) was discovered as the first member of the genus *Hepacivirus* (1) and subsequently characterized as a hepatotropic virus with high chronicity rates causing severe liver damage (2). More than 70 million people are still chronically infected with HCV worldwide, despite the availability of direct-acting antiviral (DAA) drugs with cure rates above 90% (3). The discrepancy between the availability of good therapeutic intervention strategies and the remaining high prevalence of HCV is mainly caused by underdiagnosis, uneven distribution of DAAs, and lack of protective immunity after overcoming infection (4). HCV puts patients at risk for severe liver damage, resulting in 400,000 deaths annually (4). This could be prevented with a prophylactic vaccine, which would also support the WHO’s goal of eradicating HCV by 2030. However, vaccine design remains challenging for several reasons. HCV is a highly variable virus that rapidly escapes detection by neutralizing antibodies (5–7). Conserved epitopes are protected by highly variable regions, glycosylation, or association with lipopolyproteins (8–13). Moreover, the lack of an immunocompetent animal model that supports efficient HCV propagation hinders vaccine development (14, 15).

Understanding how to outsmart HCV’s immune evasion requires knowledge of which epitopes to target or which immune pathways to activate. Characterizing immune signatures associated with HCV clearance in humans, however, remains difficult, as HCV is rarely identified in the acute phase of infection and detailed transmission history is known less often. Therefore, an immunocompetent model system that allows for controlled infection experiments is urgently required to understand correlates of immune protection.

Since 2011, HCV-like viruses were identified across a broad host range and with strict species tropism, including mammals, reptiles, and birds (16). These newly identified hepaciviruses are of particular interest with respect to the evolutionary history of the genus *Hepacivirus*, including HCV, as well as an immunocompetent surrogate model.

To date, EqHV is the closest known relative to HCV (17), sharing 35% to 65% amino acid similarity dependent on the genetic region (17). In addition, both viruses share similar virologic features, including hepatotropism, high titer RNA replication, induction of immune-mediated liver damage, interaction with mir122, lack of protective immunity, acute and chronic courses, and delayed seroconversion (18–26). Indications of liver inflammation usually start at the time of seroconversion, but even peak enzyme values often remain within the reference interval. Histopathologic changes are often subtle, including piecemeal hepatocyte necrosis and subjective mononuclear cell infiltrates (19, 20, 25), but severe hepatopathy was also described (21). Seroprevalence in horse cohorts ranges between 22% and 84% (18, 23, 27–31), with relatively low rates of EqHV RNA-positive equids indicating a high rate of self-limited infection, in contrast to HCV causing persistent infection in 80% of patients. A key feature of a surrogate model for HCV vaccine design comprises a dual infection outcome, ideally with robust and inducible chronic infections.

Equids have been experimentally inoculated with EqHV in several studies (19, 20, 25, 26, 32). However, the majority of inoculated horses developed a self-limited acute infection, and only horses 8 months of age or younger—a phase in which the equid immune system is not fully developed yet—were persistently infected (20, 22). Inoculation studies used different protocols for infection with varying inoculation volumes (1 to 500 mL) and EqHV genome equivalents (~2 × 10^4^ to 3.9 × 10^9^) (19, 20, 26). Notably, infection with high volumes of serum or high titer inoculum can lead to a strong induction of the innate immune system, supporting viral clearance.

Thus, in this study, we evaluated the minimal infectious dose of EqHV and then characterized immune responses in eight horses, aiming to understand mechanisms of EqHV clearance and persistency.

## RESULTS

### EqHV is highly infectious in horses and is linked to a dose-dependent infection outcome.

To determine the minimal infectious dose and gain insights into the dose-dependent kinetics of hepaciviral infection, we inoculated eight EqHV-naive horses with plasma from an EqHV-positive donor horse. The plasma contained 1.3 × 10^5^ EqHV RNA copies/mL and the horses were infected with varying doses of 1–2 copies to 1.3 × 10^6^ RNA copies per inoculation (Table 1).

**TABLE 1:** Details of the animals included in this study^*a*^

ID	Sex	Age (yr)	Breed	Dose (mL)	GE
D1	G	12	Austrian Warmblood	10^1^	1.3 × 10^6^
D2	F	19	Trotter	10^0^	1.3 × 10^5^
D3	F	23	Selle Français	10^–1^	1.3 × 10^4^
D4	F	13	Oldenburger Warmblood	10^–2^	1.3 × 10^3^
D5	F	12	Oldenburger Warmblood	10^–3^	1.3 × 10^2^
D6	F	25	Zangersheide	10^–4^	1.3 × 10^1^
D7	F	7	Hanoverian Warmblood	5 × 10^–5^	6.5 × 10^0^
D8	F	17	Oldenburger Warmblood	10^–5^	1.3 × 10^0^

a G, gelding; F, female; GE, genome equivalents per inoculation.

**FIG 1:**
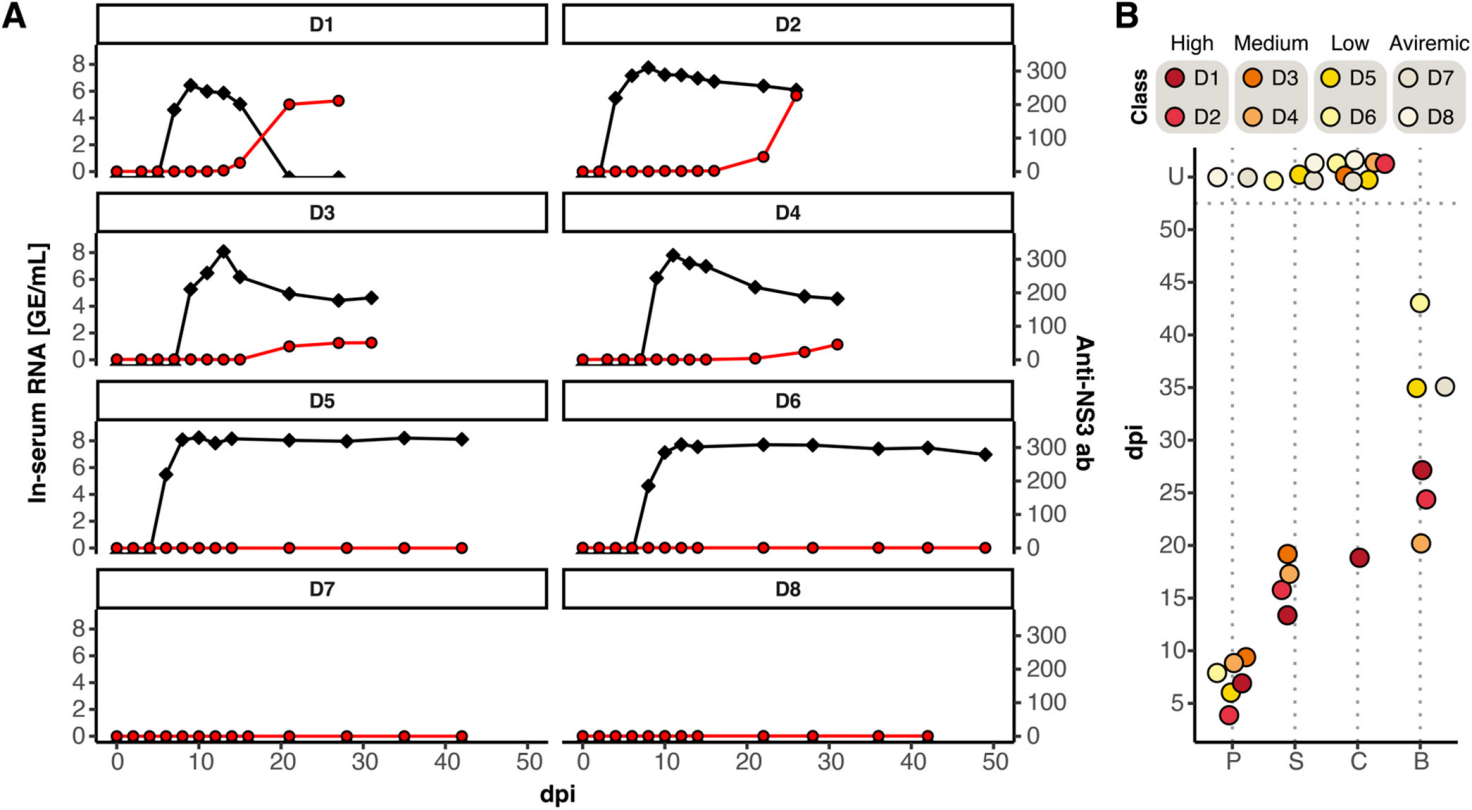
Dose-dependent equine hepacivirus (EqHV) infection in equids. (A) Viral kinetics and serology. The black line indicates viral genome equivalents per mL serum and the red line indicates normalized anti-NS3 antibody levels as fold change above background. Background is defined as three times the standard deviation from the mean of the negative control (negative plasma). (B) Experimental design and sampling time points in this study in days postinoculation (dpi). P, first time point with positive quantitative PCR (qPCR); S, time point of seroconversion (luciferase immunoprecipitation system [LIPS] 2-fold above background); C, time point of clearance; B, day of liver biopsy. If either P, S, C, or B did not occur within the monitoring time frame, time points were set to U (unknown).

Horses D1 to D6 became viremic within 4–9 days postinfection (mean 7.1 days), whereas horses D7 and D8, infected with 1–2 or 6–7 RNA copies, respectively, remained EqHV RNA-negative (Fig. 1A). Time until viremia, as well as peak viremia, did not differ between inoculation loads, reaching up to 10^8^ EqHV RNA copies/mL (Fig. 1A and B).

Seroconversion was observed in horses D1 to D4, with differences in timing and strength. In horses infected with high doses (D1 and D2), seroconversion occurred at days 13 and 16, with peak anti-NS3 antibodies levels more than 200-fold above background (Fig. 1). In contrast, seroconversion in horses inoculated with medium doses (D3 and D4) occurred slightly delayed at days 19 and 17, but anti-NS3 antibody levels were less strongly induced and were only 75- and 30-fold above background levels, respectively (Fig. 1). In those four horses, seroconversion was accompanied with decrease in viral load, but only D1 was able to clear the infection within the monitoring period. Interestingly, horses D5 and D6, which were inoculated with low doses, had similar viral kinetics compared to horses inoculated with high or medium doses, but they did not develop detectable anti-NS3 antibodies within the surveillance period (Fig. 1).

Liver enzymes were unremarkable or slightly elevated in individual horses, with no apparent relationship to infectious dose (Fig. S1). Of note, only γ-glutamyltranspeptidase (GGT), glutamate dehydrogenase (GLDH), and aspartate aminotransferase (AST) were slightly elevated. Overall, there was evidence of subclinical hepatitis of short duration only in horses D1 and D6.

**FIG 2:**
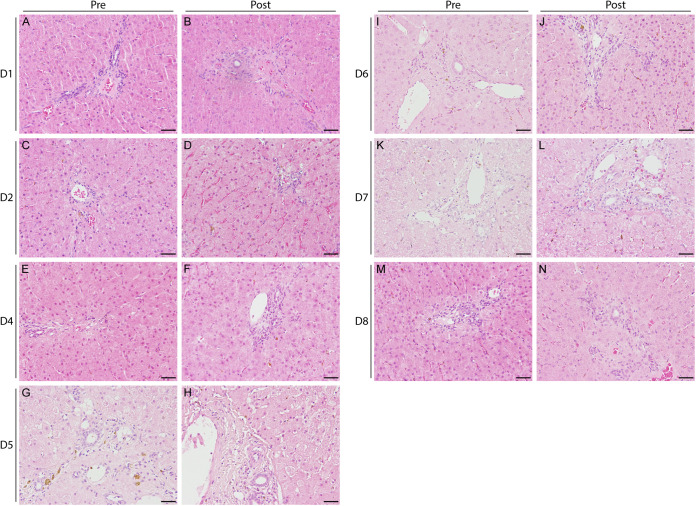
Intrahepatic histopathological changes. Representative hematoxylin and eosin staining from localizations of the liver biopsy specimens before (A, C, E, G, I, K, M) and after infection (B, D, F, H, J, L, N). The horses were infected with 1.3 × 10^6^ (D1: panels A and B), 1.3 × 10^5^ (D2: panels C and D), 1.3 × 10^3^ (D4: panels E and F), 1.3 × 10^2^ (D5: panels G and H), 1.3 × 10^1^ (D6: panels I and J), 6 to 7 × 10^0^ (D7: panels K and L) and 1 to 2 × 10^0^ (D8: panels M and N), respectively. All samples displayed minimal to mild periportally accentuated lympho-histiocytic infiltrates. Occasionally, single eosinophils or neutrophils and an intracytoplasmic, yellowish to brownish pigment in hepatocytes and Kupffer cells were present. Scale bar = 50 μm.

Liver biopsy specimens were obtained from 7 out of 8 horses (D1, D2, D4 to D8) both before inoculation and 17 to 43 (median: 35) days later. In horse D3, the liver on the right side of the chest was not visible on ultrasound. In addition, a large blood vessel traversed the left lobe of the liver at every accessible plane, preventing biopsy in both cases. Histologic examination revealed minimal to mild, multifocal, periportally accentuated, lympho-histiocytic infiltrates in all samples (Fig. 2). Additionally, single neutrophilic or eosinophilic granulocytes, depositions of yellowish-brown pigment in the cytoplasm of Kupffer cells and hepatocytes (most likely hemosiderin), and single necrotic hepatocytes (horse D5) were observed in some of the samples, independently of the time point (either or both in the initial and final sample).

### *In vivo* titration of EqHV associated with dose-dependent intrahepatic transcriptional regulation.

**FIG 3:**
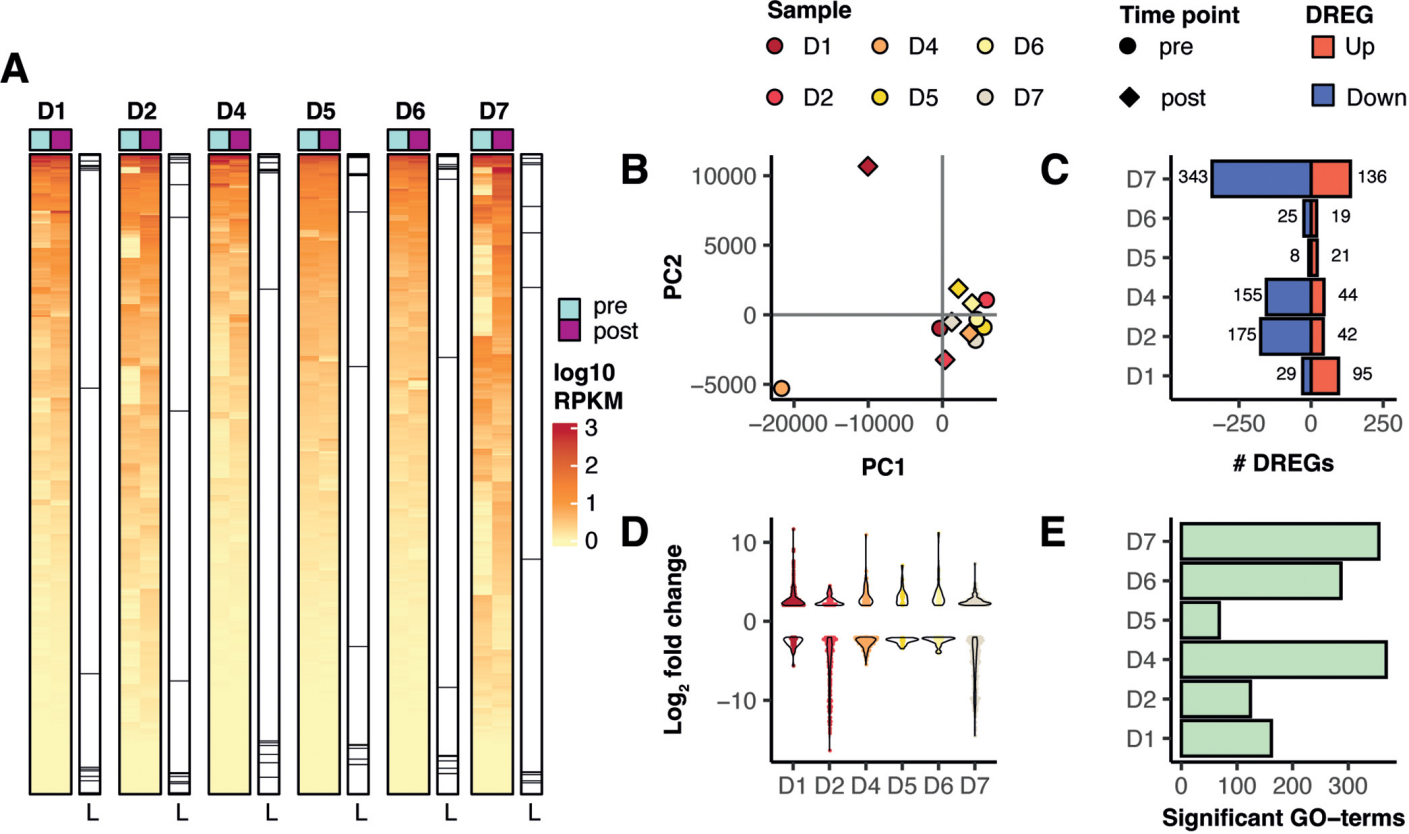
Broad transcriptional response coincides with infectious dose for productive—but not for aviremic—infection. (A) Heatmap of differentially regulated genes. Marker lanes for genes expressed in the liver (L), indicating the authenticity of liver tissue. Red colors correspond to higher expression (in log_10_ reads per kilobase million [RPKM]), while yellow corresponds to lower expression. (B) Principal-component analysis indicating differences between animals and between pre- and postinfection samples. (C) Quantification of deregulated genes (DREG, RPKM ≥ 0.5 and fold change ≥ 4). Red bars indicate upregulated genes and blue bars show downregulated genes. (D) Strength of deregulation for each animal. Dots represent each gene with significant deregulation (false-discovery rate [FDR] ≤ 0.5) and violins indicate overall distribution. (E) Number of activated gene ontology (GO)-terms (GO::BP) selected by FDR ≤ 0.05.

Liver biopsy specimens from horses D1, D2, D4, D5, D6, and D7 were collected pre- and postinfection for transcriptomics analysis (Table 1). Biopsy specimens from horse D8 were excluded due to insufficient RNA yield. All samples abundantly expressed liver markers, demonstrating the authenticity of the liver tissue (Fig. 3A). Moreover, clear differences were evident between pre- and postinfection (Fig. 3A and B). All animals had a substantial number of deregulated genes (reads per kilobase million [RPKM] ≥ 0.5 and fold change ≥ 4). Up- and downregulation was more pronounced in horses inoculated with high (D1, D2) or medium doses (D4) than in horses inoculated with low doses (D5, D6). Interestingly, horse D7, which was inoculated with only 6–7 copies of RNA and remained aviremic, had the highest number of deregulated genes (DREGs) (Fig. 3C and D). A similar pattern emerged when comparing the number of activated (false discovery rate [FDR] ≤ 0.05) gene ontology terms (GO-terms) across all animals (Fig. 3E).

**FIG 4:**
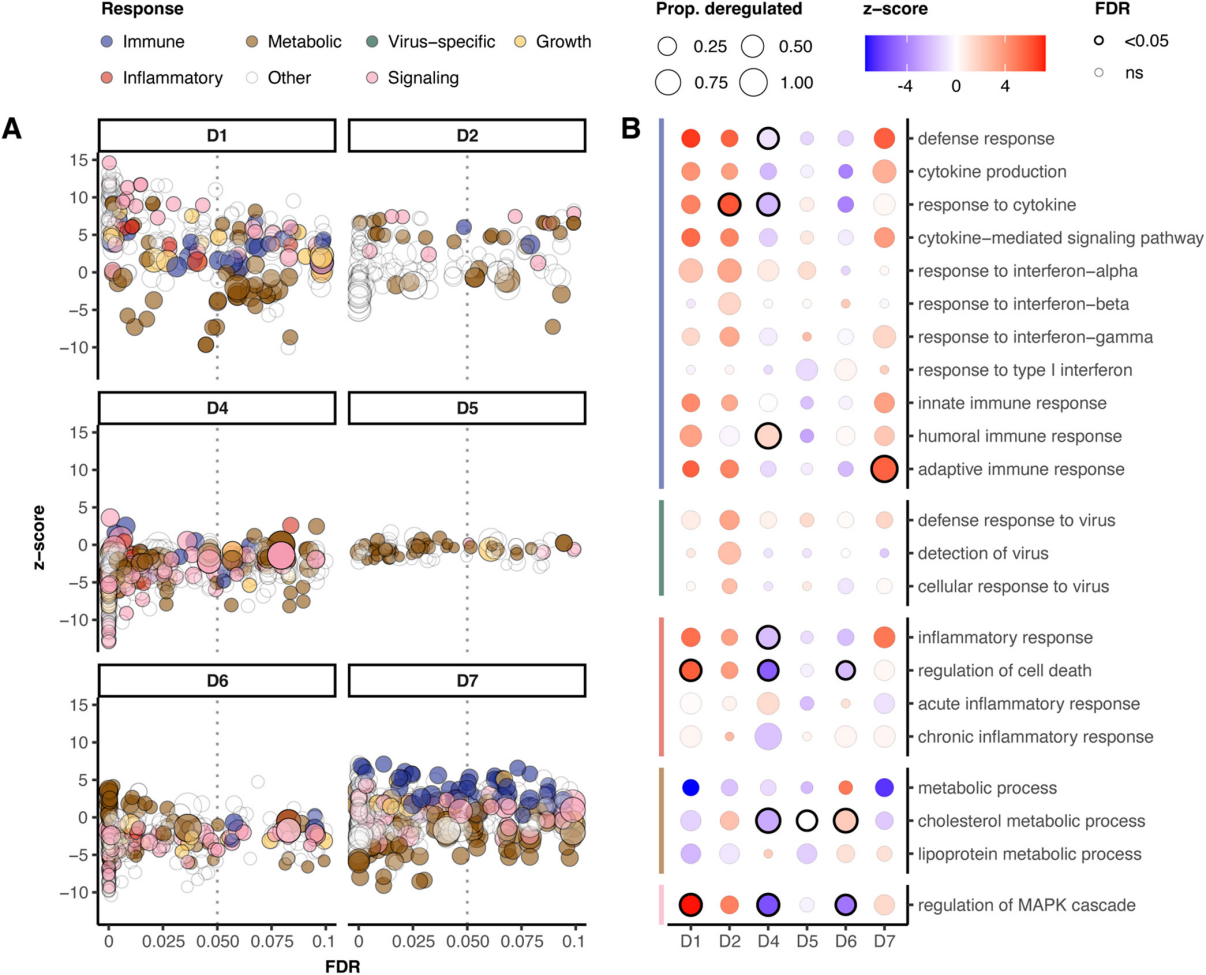
GO-term classification reveals dose-dependent immune responses. (A) Detailed view of differential activation of GO-terms. The z-score indicates the direction of gene regulation within each term, with orange indicating activation and blue deactivation. Colors show biological processes corresponding to the following terms: immune (green), metabolic (purple), virus-specific (light blue), growth (yellow), inflammation (red), and signaling (gray), and the remaining terms are transparent. Dot size indicates the number of genes differentially expressed within each term relative to unregulated genes. A border rim indicates significant deregulation (FDR ≤ 0.5). (B) Selection of GO-terms for each animal representing the same classes. Size corresponds to the size ratio, color to the z-score, and rim to significance (FDR ≤ 0.05).

To gain insight into the processes involved in infection biology, we characterized GO-terms describing biological processes. In Fig. 4A, the deregulated GO-pathways were assigned to functional classes. Consistent with previous patterns, deregulation was strongest in the high dose (D1, D2) and in the dose that did not lead to productive infection (D7). Moreover, a strong response was associated with the activation of a wide range of biological processes, including immune, antiviral, and inflammatory responses as well as cell signaling. In contrast, horse D4 exhibited few deregulated GO-pathways, which were primarily part of the term metabolic processes. We then selected GO-terms representing each class, focusing on terms related to immune, antiviral defense, and inflammatory processes (Fig. 4B). Interestingly, many immune-related terms, including responses to cytokines and IFN-α, adaptive immune response, pattern recognition pathway signaling, and defense response to virus, followed an almost dose-dependent deregulation, from strong upregulation in the high-dose infection to weaker deregulation in medium- and low-dose infections. Again, in agreement with previous results, the horse with aviremic infection (D7) showed similar deregulation to the high-dose infections. Other GO-term classes followed a similar pattern, including those associated with pathology: regulation of cell death, inflammatory responses, and metabolic processes.

In summary, the patterns of regulation of biological processes are consistent with inoculation dose and viral kinetics, explaining the prolonged viremia and lack of seroconversion in low-dose infections.

### Overlapping gene signatures of hepaciviral infection in equids and humans.

Next, we compared the signatures of gene regulation in the dose-dependently infected horses to a well-characterized open access data set from a human HCV cohort (33). Briefly, the HCV data set discriminates intrahepatic immune responses in an HCV cohort between high and low ISG response.

**FIG 5:**
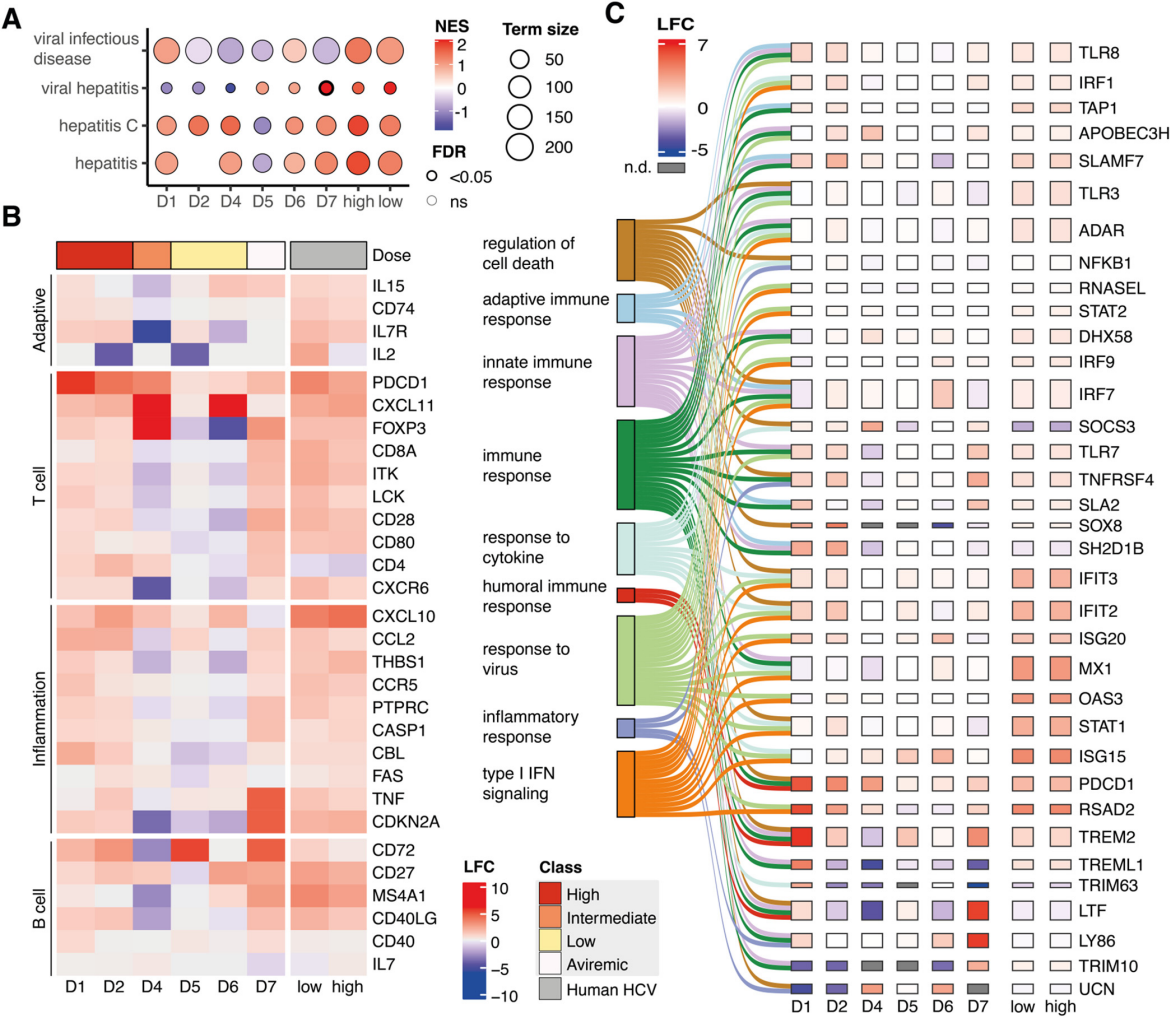
Gene signatures in inflammation, innate, and adaptive immune responses between hepaciviral infections in equids and humans. Data from human cohort were taken from Boldanova et al. (33) and are divided between low- and high-ISG response HCV-infected patients. (A) Disease ontology analysis. Dot color corresponds to the normalized enrichment score (NES), indicating whether a term is positively (orange) or negatively (blue) enriched. Dot size illustrates the number of genes differentially regulated in each term. (B) Heatmap of genes involved in disease ontology enrichment and inflammatory responses. Color corresponding to log_2_-fold change (LFC). (C) Sankey plot linking GO-terms to their corresponding genes. Gene boxes are colored according to their LFC.

We performed a disease ontology (DO) analysis to characterize expression patterns which correlate with signatures of viral hepatitis (Fig. 5A). The selected terms from Fig. 5 were all differentially enriched, indicating that there were typical signs of liver disease caused by an infectious agent. Of note, the horse cohort contained both positively and negatively enriched terms, whereas the HCV cohort had only positively enriched terms. We included the term hepatitis C, which was positively enriched in all horses except for D5. The term viral hepatitis was negatively enriched in D1, D2, and D4 and positively enriched in the other horses. This analysis revealed that terms associated with hepatitis and viral infection were enriched and differentially regulated between horses inoculated with different doses.

Followingly, we selected deregulated GO- and DO-terms, which represent immune and pathological processes, and plotted them against the fold change of a selection of deregulated genes which were included in those terms (Fig. 5B). Of note, we divided the genes into corresponding arms of the adaptive immune system. Indeed, gene expression correlated with infection dose in most cases. Upregulation was often more pronounced in high-dose infection, whereas lower doses showed a weaker deregulation. As before, the aviremic horse (D7) was an exception. Here, the transcriptional pattern showed similar deregulation to that in horses inoculated with high doses. In addition, there was dose-dependent deregulation within T and B cell regulatory genes, including *PDCD1*, *CD8A* and *CD40LG*, *CD27*, and genes involved in the inflammatory response, *CDKNA2* and *TNF* (Fig. 5B).

We then analyzed innate immune responses between the different doses and the HCV cohort connecting GO-terms with genes that contributed toward their deregulation (Fig. 5C). Common interferon-stimulated genes (ISGs) such as *RSAD2*, *ISG20*, and *IRF1* were again strongly deregulated, often in a dose-dependent manner. Similarly, the expression of pattern recognition receptors TLR3, TLR8, and TLR9, involved in the sensing of RNA viruses was deregulated in a dose-dependent fashion. Comparing gene expression profiles in the high- and low-ISG HCV cohorts, we found fold changes to be more similar between high-dose infections, aviremic infection and the high-ISG cohort. Intermediate, and low-dose EqHV infections were more similar to the low ISG cohort. Namely, these genes were all upregulated and exhibited higher fold changes in the high-ISG cohort than in the low-ISG cohort, with the exception of *PDCD1*. In addition, SOX8, a marker for human hepatocellular carcinomas, was strongly upregulated in high-dose infection and had a smaller impact in lower-dose infections; in contrast, upregulation was more pronounced in the low-ISG cohort than in the high-ISG cohort. Interestingly, *ISG15*, an interferon-stimulated gene involved in ubiquitination of newly synthesized genes, was weakly expressed in the highest-dose infection and more strongly expressed in the lower doses. Similarly, *ISG15* was also not differentially regulated in the low or high ISG cohort. Additionally, the expression of a variety of ISGs, including *MX1*, *OAS3*, and *ADAR*, was only slightly altered (Fig. 5C).

To conclude, we here examined intrahepatic transcriptional responses in horses infected with EqHV and an HCV cohort. The analysis revealed patterns consistent with infectious dose influencing transcriptomic responses for EqHV. Activation of immune and inflammatory signatures was similar for EqHV and HCV. Overall, our experiments provide detailed insights into early intrahepatic immune responses which may contribute to clearance and viral persistance.

## DISCUSSION

Here, we investigated the minimal infectious dose of EqHV in eight naive horses, characterizing the infection kinetics and the transcriptional profile. Inoculation doses ranged from 1–2 copies to 1.3 × 10^6^ RNA copies, and the minimum infectious dose which caused viremia was 13 RNA copies in our experiments. Interestingly, high-dose inoculations resulted in early seroconversion, whereas horses infected with low doses did not seroconvert during the study period. Similarly, intrahepatic immune responses were more strongly deregulated in high-dose infections. This suggests that the inoculation dose may contribute to immune detection and possibly to infection outcome.

In previous EqHV infection studies, most animals were acutely infected and prolonged viremia was rarely observed. Inoculation techniques have varied, with inoculation volumes ranging from 1 to 500 mL serum or plasma, corresponding to approximately 2 × 10^4^ to 3.9 × 10^9^ RNA copies per infection (19, 20, 26, 32). In addition, inoculation with serum or plasma often has the disadvantage that the host immune system, especially at high doses, could trigger an early and strong immune response that contributes to viral clearance. To circumvent this, the viral inoculum can be ultracentrifuged to remove serum components, or *in vitro*-transcribed RNA can be injected intrahepatically (25, 32). In our study, we inoculated with small amounts of plasma in the low-dose infections, which may prevent strong immune activation by plasma components and thus contribute to sustained viral replication. Of note, many other factors could contribute to the duration of infection, including viral population dynamics, virus genotype, transmission mode, and host genetics, all of which have been shown to be important for HCV (34–36). Moreover, EqHV was shown to be less heterogenic than HCV and lack the hypervariable region 1 (HVR1) at the E2 N-terminus, an important feature for HCV transition into persistence (37).

The minimum infectious dose of 13 EqHV RNA copies estimated here was sufficient to cause high-titer infection, while 7 or fewer RNA copies were insufficient to establish an infection. This is probably consistent with needlestick reports in HCV-infected patients, where a trace amount of blood is sufficient to cause infection (38–40). Furthermore, this is similar to the minimal doses described for HCV in chimpanzees (41, 42) and Norway rat hepacivirus (NRHV) in rats (43), which were 20 to 100 and 10 RNA copies per inoculation, respectively. For NRHV, virus kinetics in C57BL/6J and BALB/c mice were not affected by the inoculation load, which ranged from 10 to 10^4^ RNA copies. In all cases, the virus was cleared within 5 weeks postinfection (43). However, host restriction may have played an important role here since rats, not mice, are the natural hosts for RHV. For HCV in chimpanzees, two studies have characterized the minimum infectious dose required for transmission. Katayama et al. (42) successfully infected two chimpanzees with 20 HCV genome equivalents. In both, viremia was detected between 6 and 12 weeks, but only one seroconverted at week 24. Two other chimpanzees in this study, inoculated with 7 × 10^6^ copies, developed anti-HCV within 10 to 11 weeks, consistent with literature (42, 44, 45). In Shata et al. (41), the minimum infectious dose was 100 copies per inoculation, while lower doses were insufficient to cause viremia but were efficient in stimulating HCV-specific immune responses, a finding which was in agreement with previous reports (39, 46, 47). This may explain the observations in horse D7, which remained RNA-negative but elicited a strong immune system induction. Moreover, early and stronger immune responses have been observed to be associated with HCV clearance (41). To rule out infection with other viral pathogens, we examined serum samples from horse D7 before and after infection (matching to liver biopsy samples). No differences in antibody levels were detected for tick-borne encephalitis virus, West Nile virus, borna virus, equine influenza A-1 and 2 viruses, or equine herpesviruses 1 to 4 (Fig. S2). In addition, we analyzed NGS sequencing reads that did not match the equine reference genome for the presence of viral signatures. Consistent with the serologic assay, no increase in viral reads was observed between the two time points. Therefore, it is unlikely that horse D7 acquired a viral infection during the sampling period that could be responsible for strong immune activation (Fig. S2).

In this study, seroconversion was detectable only for high- (D1, D2) and medium- (D4) dose infections, while it was absent in low-dose inoculations (D5, D6). Similarly, in rare cases, chimpanzees infected with HCV have been reported to remain seronegative for prolonged periods of up to 5 years (42, 48, 49). Accordingly, HCV RNA-positive but anti-HCV-negative HCV-infected patients have been described (50–54). In both HCV-infected chimpanzees and humans, clearance can be achieved even in the absence of anti-HCV antibodies, highlighting the importance of innate immune responses and other effector cells (47). On the other hand, adaptive immune responses were shown to have a strong effect on virus replication. In this and previous studies, virus copy numbers decrease with the appearance of anti-EqHV antibodies (18–20, 26, 32). Similarly, HCV in chimpanzees and humans is often eliminated with co-occurrence of strong adaptive immune responses (38, 55, 56). Indeed, we were able to show that markers for the activation of innate immune responses, including T and B-cell activation, were more abundantly expressed in high- than in low-dose infections. Interestingly, foals aged 8 months or younger often fail to clear EqHV, likely due to the immature immune system’s inability to elicit an efficient adaptive immune response (20, 22). Furthermore, foals often remain EqHV RNA-positive after extended periods during which their immune system should be fully developed (20, 22). This is, to some degree, consistent with the sustained viremia in low-dose infections in our study, as EqHV may be able to achieve sustained replication after establishing a viral population and bypassing initial immune responses. Thus, the EqHV surrogate model might be useful for future experiments aimed at understanding the mechanisms of hepacivirus immune evasion and persistence.

Of note, in our study we were not able to monitor animals for longer than 50 days, so there is a possibility of delayed seroconversion after monitoring. In addition, we measured only anti-NS3 antibodies, so it is possible that antibodies are directed against other structural proteins. Because this project was designed as a proof-of-principle study to evaluate the minimal dose of infection, we were only able to test each dose once.

Despite these limitations, clear trends were evident in the intrahepatic transcriptome which were consistent with serological observations. Gene expression signatures showed strong upregulation of immune and inflammatory processes in high-dose inoculation compared to that in lower doses. The pathways involved include cytokine signaling, innate and adaptive immune system processes, and inflammation markers. Strong upregulation of these pathways has been associated with clearance, as has also been reported for HCV in chimpanzees and humans, while their absence can promote persistence (55–58). In general, studies of early immune responses in humans remain challenging because HCV often remains undetected until progression into chronicity and accumulation of liver damage. Additionally, comparison of intrahepatic antiviral defenses with biopsy specimens before infection is nearly impossible in humans.

Another limitation of our study was the time points of the liver biopsy samples, which were taken between days 20 and 43. At this time, animals were in different stages of infection, with some animals already seroconverted and others not. Nevertheless, intrahepatic gene expression analysis enabled unique insights into virus-host dynamics during the acute phase of hepaciviral infection in a dose-dependent manner.

In conclusion, an animal model which allows for controlled and robust hepacivirus infection may help to unravel rare instances of silent and aviremic infections. An immunocompetent surrogate model such as EqHV in horses may thus pave the way to understanding the mechanisms by which hepaciviruses suppress immune recognition and identifying correlates of protection in the absence of neutralizing antibodies. Both of these could be useful for vaccine development.

## MATERIALS AND METHODS

### Animals.

Eight horses (Table 1) were included in the study, which was conducted at the Clinic for Horses of the University of Veterinary Medicine Hannover from March 2019 to February 2021. The horses were housed in isolation with daily turnout on a sand paddock from 1 week prior to infection. Clinical examinations were performed daily. Immunocompetence was initially determined by the absence of clinically detectable infectious disease and inconspicuous routine hematology. The State Office for Consumer Protection and Food Safety (LAVES) approved the study in accordance with the German Animal Welfare Law (file no. 33.8-42502-04-17/2594).

### Infection protocol.

Serum samples for detection of EqHV RNA and anti-NS3 antibodies were obtained from all horses just before their inclusion in the trial to ensure that they were naive to EqHV. On the day of infection, a blood sample was drawn before the horses were sedated in stocks. After aseptic preparation of the surgical site, a trained veterinarian took liver biopsy samples under ultrasonographic guidance and local infiltration anesthesia with lidocaine. Biopsy samples were either put into formalin (used for histology) or immediately frozen in liquid nitrogen (used for next-generation sequencing [NGS]) before being stored at −80°C. After sampling, the horses were administered 1.1 mg/kg body weight flunixin-meglumin intravenously once (Flunidol RPS 50 mg/mL; CP-Pharma Handelsgesellschaft GmbH, Burgdorf, Germany).

The horses were included in the study in cohorts of two. The first two horses were infected with 1 and 10 mL plasma, respectively. The infectious plasma used throughout the study came from a single donor with 1.3 × 10^5^ RNA copies per mL (±3.2 × 10^4^) (19). The doses for subsequent cohorts were chosen iteratively depending on the outcomes of the infections in previous cohorts, as detailed in Table 1. The infectious plasma was administered in 100 mL of saline using an infusion pump over 10 min to monitor the horses for any adverse reactions.

### Sample acquisition.

From the day of infection, a blood sample was taken every other day for 2 weeks and then once a week until EqHV clearance. Routine hematology (Automated Hematology Analyzer XP-300; Sysmex Deutschland GmbH, Norderstedt, Germany) parameters, as well as liver enzymes and specific substrates (cobas c311 analyzer; Roche Diagnostics Deutschland GmbH, Mannheim, Germany) were determined on the day of sampling. The serum samples were centrifuged at 1,000 × *g* for 10 min, aliquoted, and stored at −80°C until further analyses. A second biopsy specimen was taken 17 to 43 days (median: 35) after infection following the procedure described above. Formalin-fixed biopsy specimens were paraffin-embedded, cut at 2 to 4 μm, and stained with hematoxylin and eosin for histological analysis.

### Detection of anti-EqHV NS3 antibodies.

Anti-EqHV NS3 antibodies were semi-quantitatively measured by luciferase immunoprecipitation system (LIPS) as previously described (27). Serum samples diluted 1:10 in buffer A (50 mM Tris [pH 7.5], 100 mM NaCl, 5 mM MGCL2, and 1% Triton X-100) were measured in duplicates. Luciferase-NS3 fusion antigen (10^7^ relative light units) was mixed in 40 μL buffer A and then added to 10 μL of diluted sera. The mixture was shaken at room temperature for 1 h. Antigen was precipitated using 30% Ultralink protein A/G beads (Pierce Biotechnology, Rockford, IL) added to a 96-well filter HTS plate (Millipore, Bedford, MA). Afterwards, 100 μL of the RLUC-NS3 antigen serum mixture was added and incubated for 1 h shaking at room temperature. The filter plate was then washed with phosphate-buffered saline on a vacuum plate washer. Antigen-precipitation was quantified by adding 100 μL coelenterazine (PJK GmbH, Kleinblittersdorf, Germany) on a Berthold LB960 Centro XS3 plate luminometer (Berthold, Freiburg, Germany). The threshold for NS3-antibody positive samples was set at three standard deviations above the average of the negative controls.

### Viral RNA quantification.

Viral genomic RNA was isolated from plasma using the High-Pure Viral RNA kit (Roche, Mannheim, Germany) according to the manufacturer’s manual. cDNA was generated using random hexamer primers (Prime Script RT Master Mix kit; TaKaRa Bio, Shiga, Japan). Quantification of EqHV genome copies was performed via the SYBR Premix Ex Taq II kit (TaKaRa Bio, Shiga, Japan) using previously published primer pairs targeting the 5′ untranslated region (UTR): GAGGGAGCTGRAATTCGTGAA, GCAAGCATCCTATCAGACCGT (27). Calculation of viral genome quantities was done using *in vitro*-transcribed RNA as standard containing the 5′ UTR of EqHV isolate NPHV-NZP-1 (JQ434001).

### RNA-seq analysis.

Snap-frozen liver biopsy specimens were used for RNA isolation and NGS. Briefly, RNA was isolated from mechanically homogenized liver biopsy specimens using the Machery Nagel RNA extraction kit according to the manufacturer’s instructions. RNA quality was accessed via gel electrophoresis. Quality and integrity of total RNA was controlled on a 5200 Fragment Analyzer System (Agilent Technologies). The RNA sequencing library was generated from 500 ng total RNA using Dynabeads mRNA DIRECT Micro Purification kit (Thermo Fisher Scientific, Waltham, MA) for mRNA purification, followed by NEBNext Ultra II Directional RNA Library Prep kit (New England BioLabs, Ipswich, MA), both according to the manufacturer’s protocols. The libraries were treated with Illumina Free Adapter Blocking and sequenced on Illumina NovaSeq 6000 using the NovaSeq 6000 S1 reagent kit (100 cycles, paired-end run 2 × 50 bp), with an average of 5 × 10^7^ reads per RNA sample. Raw reads were quality-trimmed and mapped to the equine reference genome (EquCap3.0) using CLC Workbench 2020. Data were visualized in R using the following packages: tidyverse, ggplot2, ggpubr, GO-plot, clusterprofiler, DOSE, ggsankey, and complex heatmap. The RNAseq data discussed in this publication have been deposited in National Center for Biotechnology Information’s Gene Expression Omnibus (GEO) and are accessible through GEO Series accession number GSE211197.
