# Supplementary material for: Dose-Dependent Hepacivirus Infection Reveals Linkage between Infectious Dose and Immune Response
Source: Microbiol Spectr. 2022 Aug 22;10(5):e01686-22. doi: 10.1128/spectrum.01686-22 (PMC9602444; doi:10.1128/spectrum.01686-22)
Supplement: Supplemental file 1 — Fig. S1 and S2. Download spectrum.01686-22-s0001.pdf, PDF file, 0.4 MB [file spectrum.01686-22-s0001.pdf]

Supplementary Information to

Dose-dependent Hepacivirus infection reveals linkage between infectious dose and immune response

André Gömer[1, 2 *], Julien Delaroqce[3 *], Christina Puff[4], Maximilian K. Nocke[1], Birthe Reinecke[5], Wolfgang Baumgärtner[4], Jessika M.V. Cavalleri[6], Karsten Feige[3], Eike Steinmann[1 **], Daniel Todt[1, 7 **]

[1]Department of Molecular and Medical Virology, Ruhr University Bochum, Bochum, Germany
[2]Institute of Virology, University of Veterinary Medicine Hannover, Hannover, Germany
[3]Clinic for Horses, University of Veterinary Medicine Hanover, Hannover, Germany
[4]Department of pathology, University of Veterinary Medicine Hanover, Hannover, Germany
[5]Institute of Experimental Virology, TWINCORE Centre for Experimental and Clinical Infection Research, Hannover, Germany
[6]Clinical section of equine internal medicine, Department of Companion Animals and Horses, University of Veterinary Medicine Vienna, Vienna, Austria
[7]European Virus Bioinformatics Center (EVBC), Jena, Germany

[*]Contributed equally

[**]Corresponding authors

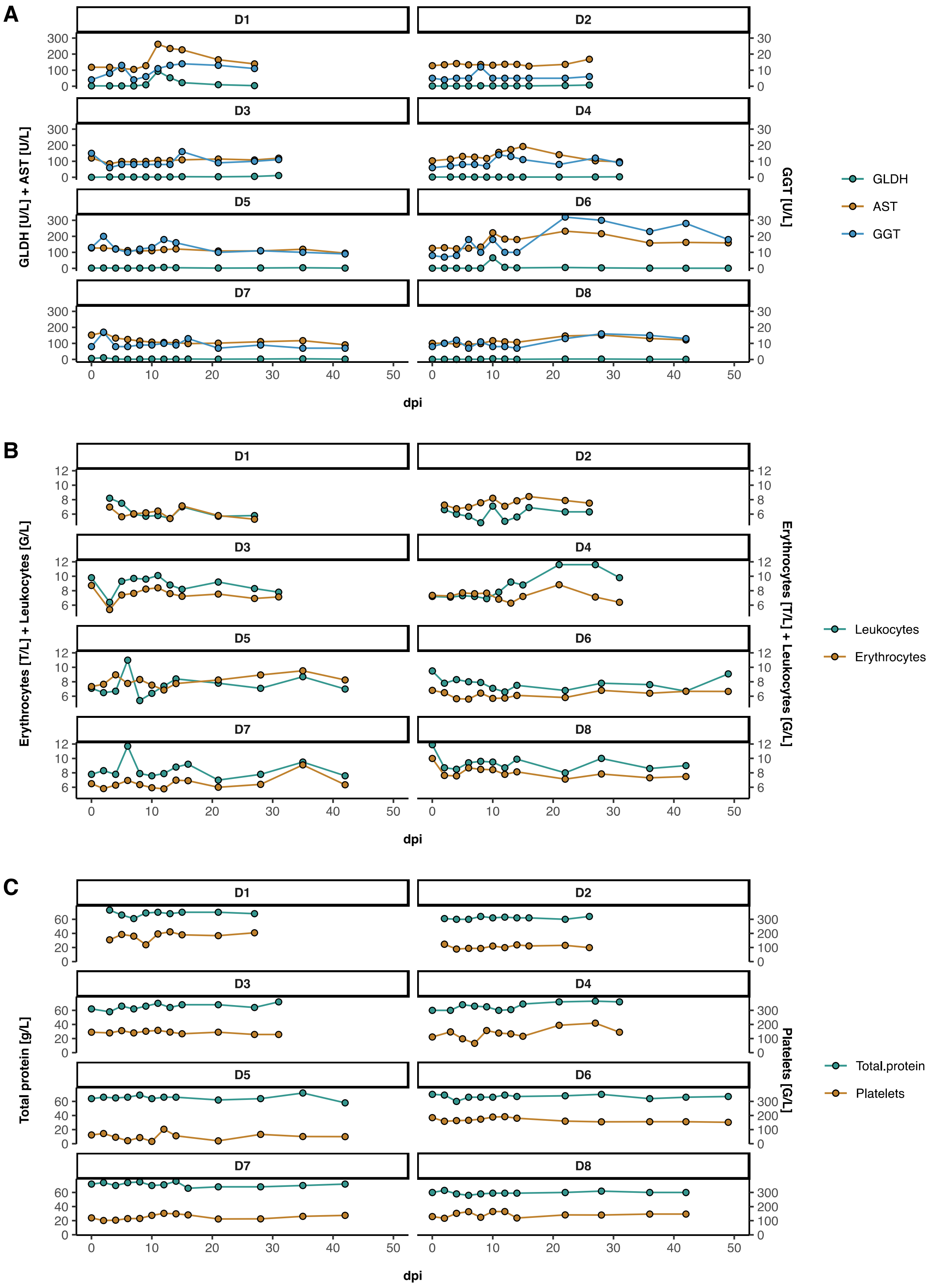
A
D1
D2
D3
D4
D5
D6
D7
D8
GLDH [U/L] + AST [U/L]
GGT [U/L]
300
200
100
0
30
20
10
0
0
10
20
30
40
50
dpi
GLDH
AST
GGT
B
D1
D2
D3
D4
D5
D6
D7
D8
Erythrocytes [T/L] + Leukocytes [G/L]
12
10
8
6
Leukocytes
Erythrocytes
C
D1
D2
D3
D4
D5
D6
D7
D8
Total protein [g/L]
Platelets [G/L]
60
40
20
0
300
200
100
Total.protein
Platelets

D

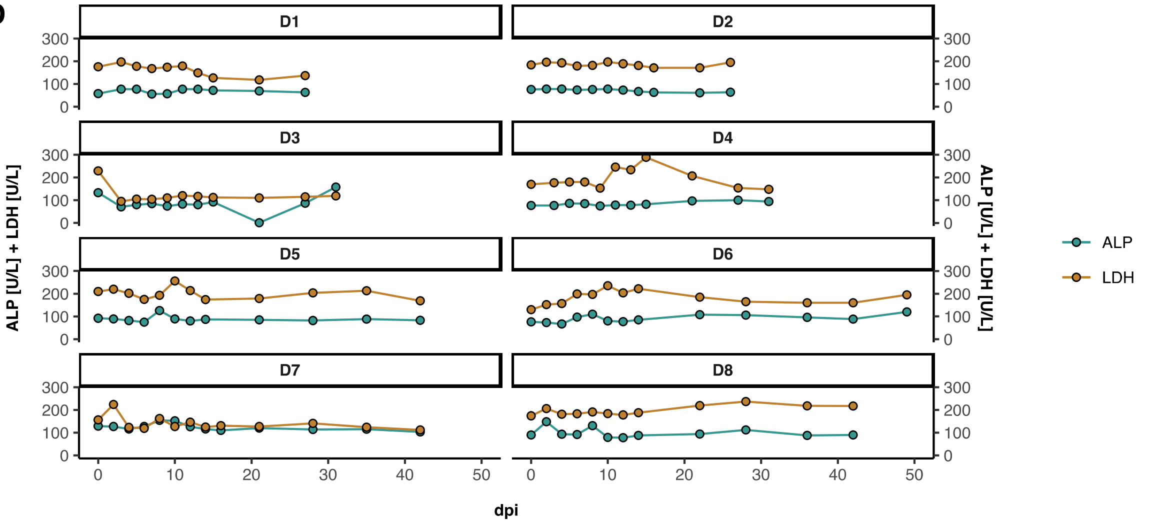
D1
D2
D3
D4
D5
D6
D7
D8
ALP [U/L] + LDH [U/L]
dpi
ALP
LDH

E

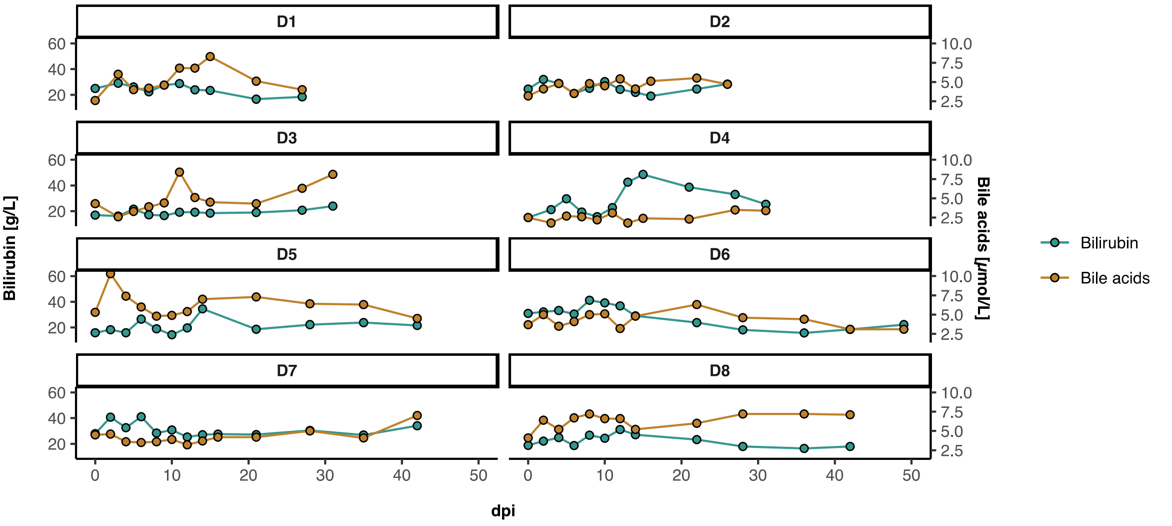
D1
D2
D3
D4
D5
D6
D7
D8
Bilirubin [g/L]
Bile acids [µmol/L]
dpi
Bilirubin
Bile acids

F

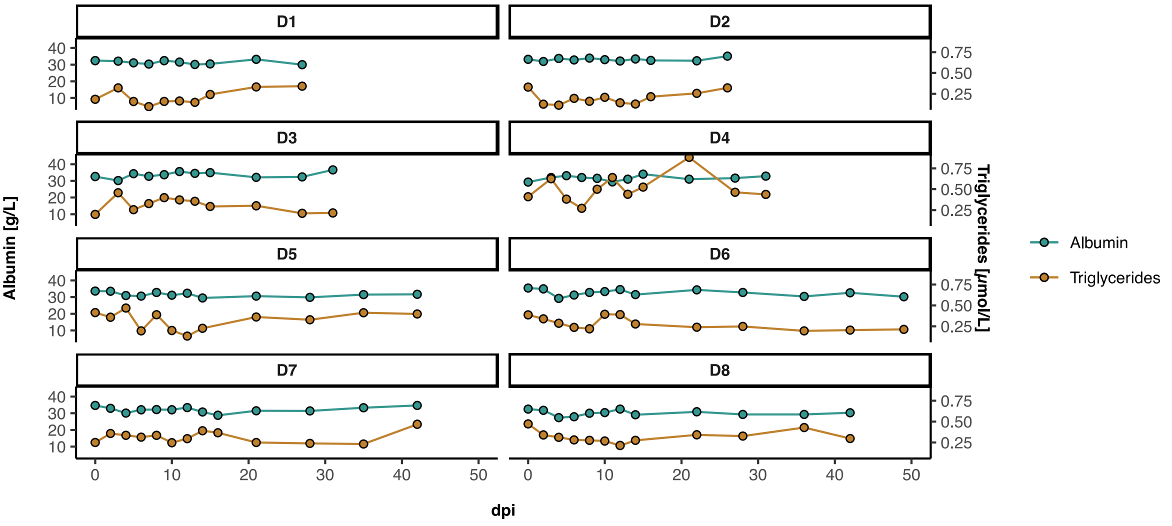
D1
D2
D3
D4
D5
D6
D7
D8
Albumin [g/L]
Triglycerides [µmol/L]
dpi
Albumin
Triglycerides

Figure S1: Course of clinical parameters. Relevant biomarkers were monitored in longitudinally drawn serum samples of study horses D1-D8 (**A-F**). GLDH (glutamatdehydrogenase), AST (aspartate aminotransferase), GGT (Gamma-glutamyl Transferase), ALP (Alkaline phosphatase), LDH (Lactate Dehydrogenase).

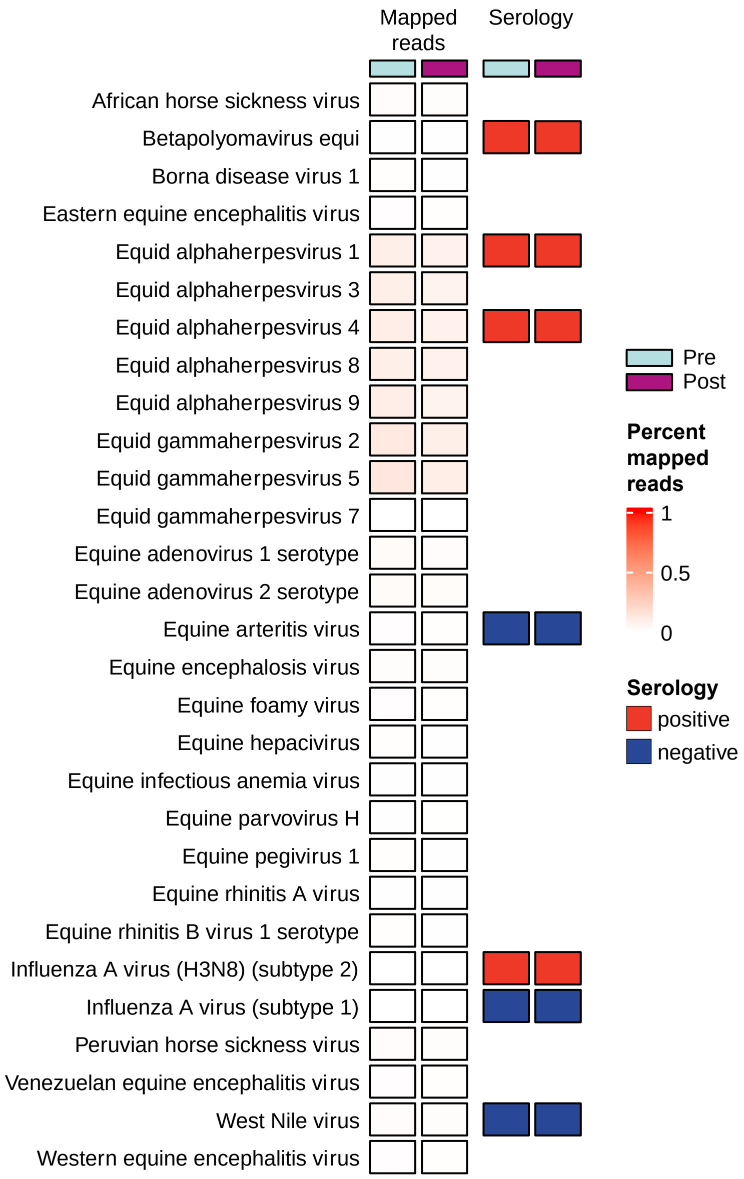

Figure S2: Analysis of viral signatures in horse D7. Serum samples drawn from horse D7 before and after infection (matching to liver biopsies) were examined for the presence of antibodies to typical equine viral pathogens. Antibody levels did not differ in any of the samples analyzed before and after inoculation with EqHV positive serum. Panel included tick-borne encephalitis virus, West Nile virus, equine influenza virus A 1/2, Borna virus, or equine herpes virus 1-4. In addition, we analyzed sequencing reads that did not match the equine reference genome for the presence of equine viruses. Consistent with the serological assay, no increase in viral reads was detected between the two time points. Analysis was performed using an in-house Julia pipeline (version 1.7.3). Briefly, the NCBI viral sequencing database (taxid 10239) was accessed to download reference sequencing files via Entrez Direct (version 16.2) which were filtered for equine viruses. Due to lacking reference

information for equine influenza virus, the viral polymerase sequence was used as a reference (KJ579963, OK559491). All sequencing reads not mapping to the horse reference genome were aligned to the reference virus database (Blast version 2.12.0). Hits were filtered for 85% identity and an alignment length of at least 20 nucleotides.
